# Pandemic (H1N1) 2009, Abu Dhabi, United Arab Emirates, May 2009–March 2010

**DOI:** 10.3201/eid1702.101007

**Published:** 2011-02

**Authors:** Gulfaraz Khan, Jamal Al-Mutawa, Muhammad Jawad Hashim

**Affiliations:** Author affiliations: United Arab Emirates University, Al Ain, United Arab Emirates (G. Khan, M.J. Hashim);; Health Authority Abu Dhabi, Al Ain (J. Al-Mutawa)

**Keywords:** Influenza, viruses, Abu Dhabi, pandemic, H1N1, respiratory infections, United Arab Emirates, dispatch

## Abstract

To ascertain characteristics of pandemic (H1N1) 2009 virus infection, we reviewed medical records for all suspected or confirmed cases reported in Abu Dhabi during May 2009–March 2010. Overall case-fatality rate was 1.4/100,000 population. Most patients who died had ≥1 risk factor, and female decedents were considerably younger than male decedents.

The outbreak of pandemic (H1N1) 2009 influenza virus was first noted in Mexico in March 2009 (*1*) but quickly spread worldwide. On June 11, 2009, the World Health Organization declared the first influenza pandemic in >40 years, triggering governments around the world to make pandemic (H1N1) 2009 a top public health priority (*2*). Although numerous published studies from around the world have described experiences with the pandemic, few have been from the Middle East. In this study, we present data from Abu Dhabi, the largest of the 7 states in the United Arab Emirates. Abu Dhabi is also the country’s capital and has a population of ≈2 million (*3*).

## The Study

By May 1, 2009, Abu Dhabi had procedures in place for reporting suspected or confirmed cases of pandemic (H1N1) 2009 (*4*). The state government made reporting mandatory, and data were recorded by Health Authority Abu Dhabi (HAAD). All health care facilities in Abu Dhabi were provided with the case definition of pandemic (H1N1) 2009 virus infection along with reporting guidelines (revised September 8, 2009) (*4*). Briefly, influenza-like illness (ILI) was defined as fever (>37.8°C) with cough and/or sore throat in the absence of known causes other than influenza. Pandemic (H1N1) 2009 was confirmed by using real-time reverse transcription–PCR according to protocol (*5*). Laboratory testing was recommended only for patients with severe illness (ILI with signs such as hypotension, dyspnea, tachypnea, abnormal radiographic appearance of the lungs) or patients with mild illness who had risk factors (e.g., pregnancy, age <5 years, chronic disease). All patients who had symptoms of influenza but negative test results by PCR for pandemic (H1N1) 2009 or who were not tested were grouped into the ILI category for statistical analysis.

Data was analyzed using PASW Statistics version 18 (SPSS Inc, Chicago, IL, USA). One-way analysis of variance was used to compare differences in mean age between the 3 groups (ILI, confirmed pandemic [H1N1] 2009 infections, pandemic [H1N1] 2009–associated deaths) and the χ^2^ test (2-sided) to compare gender and national origin of patients.

**Figure 1:**
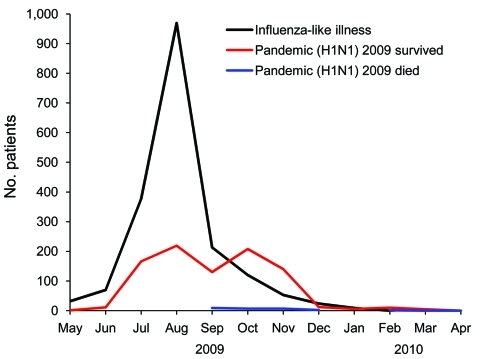
Distribution of cases of influenza-like illness (ILI), laboratory confirmed-pandemic (H1N1) 2009 in patients who survived, and pandemic (H1N1) 2009 in patients who died, Abu Dhabi, United Arab Emirates, May 1, 2009–March 23, 2010. Of the 2,806 cases reported to Health Authority Abu Dhabi, 1,872 were ILI (pandemic [H1N1] 2009 negative or status unknown), 908 were confirmed pandemic (H1N1) 2009 infections in patients who survived, and 26 were pandemic (H1N1) 2009 infections in patients who died. Patients with ILI and survivors of confirmed pandemic (H1N1) 2009 are plotted by date patient first sought care. Pandemic (H1N1) 2009 fatalities are plotted by date of death.

From May 1, 2009, through March 23, 2010, a total of 2,806 patients with confirmed or suspected pandemic (H1N1) 2009 infection were reported to HAAD. The first ILI case was recorded on May 3; the number of cases peaked in August (Figure 1). The first laboratory-confirmed case occurred on May 20, with the first pandemic (H1N1) 2009–associated death on September 1. Laboratory-confirmed pandemic (H1N1) 2009 cases showed a bimodal distribution, with the first peak in August and the second peak in October (Figure 1). The lack of a second peak in the ILI group is probably due to the change in testing recommendations issued by HAAD on September 8 (testing only patients with severe illness or with risk factors).

**Figure 2:**
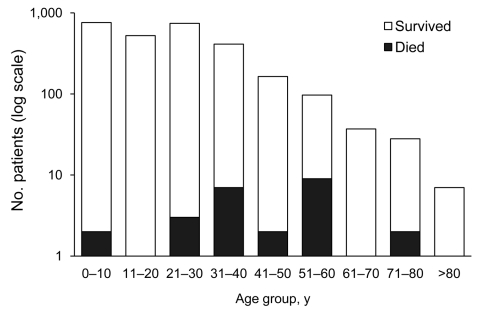
Age group distribution of patients with influenza-like illness and laboratory-confirmed pandemic (H1N1) 2009 infection, Abu Dhabi, United Arab Emirates, May 1, 2009–March 23, 2010.

Of the 2,806 patients reported, 1,872 (67%) had ILI, 908 (32%) had laboratory-confirmed pandemic (H1N1) 2009 infection and survived, and 26 (0.9%) had pandemic (H1N1) 2009 infection and died (Table 1). Of the 2,806 patients, 60% (1,679) were male; the preponderance of male patients most likely reflects the substantially higher population of male than female residents in Abu Dhabi (*3*). Of the 1,872 patients with ILI, 646 had laboratory-confirmed negative results for pandemic (H1N1) 2009; the remaining patients were not tested. Almost half (439/896, or 49%) of all laboratory-confirmed cases occurred in children and young adults <20 years of age (Figure 2). For 12 laboratory-confirmed cases, the precise age of the patient was not known. Most (21/26, 81%) decedents were 21–60 years of age; 1 reason may be that the overall population of Abu Dhabi is skewed toward younger age groups. Men who died of pandemic (H1N1) 2009 were significantly older (mean age 52.9 years, 95% confidence interval [CI] 44.0–61.7) than their female counterparts (mean age 31.5 years, 95% CI 18.9–44.1; Mann-Whitney U test, p = 0.007) (Table 2). However, these findings have to be interpreted with caution because our sample of patient deaths is small.

**Table 1:** Number of cases of influenza-like illness, laboratory-confirmed pandemic (H1N1) 2009, and pandemic (H1N1) 2009– associated deaths, Abu Dhabi, United Arab Emirates, May 1, 2009–March 23, 2010*

Illness	Total no. cases	Incidence†	Mean age, y		
			All patients	Male patients	Female patients
Influenza-like illness	1,872	97.7	23.0	22.5	23.7
Pandemic (H1N1) 2009					
Survived	908	47.4	21.6	20.7	22.7
Died	26	1.4	43.8	52.9	31.5

*Based on Heath Authority Abu Dhabi statistics for 2009 (*3*). The emirate of Abu Dhabi has a total population of 1,915,903. †Per 100,000 population.

**Table 2:** Characteristics of 26 patients who died of pandemic (H1N1) 2009 infection, Abu Dhabi, United Arab Emirates, May 1, 2009–March 23, 2010

Characteristic	Value
Gender, no. (%)	
M	15 (59.7)
F	11 (42.3)
Nationality, no. (%)	
United Arab Emirates	12 (46.2)
Expatriates	14 (53.8)
Age, y	
Mean	43.8
Median	47.0
Range	0.67–83.00
Signs and symptoms, no. (%)	
Fever	19 (73.1)
Cough	14 (53.8)
Breathing difficulty	14 (53.8)
Other,* with or without above symptoms	17 (65.4)
Underlying conditions, no. (%)	
Pregnancy	6 (54.5)
Diabetes	9 (34.6)
Malignancy	7 (26.9)
Cardio/cerebrovascular disease	2 (7.7)
Hypertension	5 (19.2)
Asthma	2 (7.7)
Other	4 (15.4)
Not recorded	6 (23.1)
Duration of oseltamivir treatment, d†	
Mean	8.3
Median	5.0
Range	2–28
Time from laboratory confirmation of pandemic (H1N1) 2009 infection to start of oseltamivir treatment, d‡	
Mean	−3.2
Median	−2.0
Range	−16 to 2
Duration from hospitalization to death, d	
Mean	27.5
Median	21
Range	1–86

*Sore throat, lung infiltration, diarrhea, headache, chest pain, abdominal pain. †Although all patients with pandemic (H1N1) 2009 were treated with oseltamivir, the exact duration of treatment was known for only 12. ‡The exact date of laboratory confirmation of pandemic (H1N1) 2009 and start of oseltamivir treatment was known for only 21 of the 26 patients.

Abu Dhabi has a high expatriate population. According to HAAD 2009 statistics (*3*), 78.8% of the population consists of persons who are not citizens of the United Arab Emirates. Patients in our study represented >50 different nationalities, the top 5 being Emirati, Indian, Filipino, Egyptian, and Pakistani. To have sufficient numbers for a meaningful statistical analysis, we grouped all reported cases into United Arab Emirate nationals (n = 1,708) or expatriates (n = 1,098). Analysis of these 2 groups showed no significant age difference (1-way analysis of variance, p = 0.357) between the Emiratis and the expatriates in terms of ILI, pandemic (H1N1) 2009 survivors, and pandemic (H1N1) 2009 decedents.

Of the 26 decedents, 15 were male; 12 were United Arab Emirate nationals, and 14 were expatriates. Calculating case-fatality rates (CFR) with laboratory-confirmed cases as the denominator is, in this type of study, inaccurate and misleading. Because not all persons with symptoms seek medical attention or are tested, pandemic (H1N1) 2009–confirmed cases are likely to be underestimated and CFR, in turn, to be grossly overestimated (*6**,**7*). We chose to represent mortality estimates per 100,000 persons because the number of fatal cases and the population are accurately known. This information gave an estimated CFR of 1.4 deaths per 100,000 persons. The mean age of decedents was 43.8 years compared with 21.6 years for persons with laboratory-confirmed pandemic (H1N1) 2009 infection who survived (1-way analysis of variance, p<0.01). The most common initial symptoms were fever, cough, and breathing difficulty (Table 2). All patients with pandemic (H1N1) 2009 who died received oseltamivir; however, complete details of antiviral treatment were available for only 21/26 cases. For 20 patients, treatment was started before or on the day of laboratory confirmation (mean −3.2 days). Mean duration of antiviral treatment was 8.3 days (range 2–28 days). Most (20/26; 77%) patients with pandemic (H1N1) 2009 who died had >1 underlying risk factor (*8*), most commonly pregnancy, diabetes, malignancy, and hypertension. Twelve decedents each had 1 risk factor, 3 had 2, 3 had 3, and 2 had 4. Mean duration from hospital admission to death was 27.5 days (Table 2).

Ages of decedents with pandemic (H1N1) 2009 infection differed significantly by gender; female patients were considerably younger (mean 31.5 years) than male patients (mean 52.9 years). Even after excluding pregnant women from the equation (*9*), female decedents remained significantly younger than male decedents. In contrast, ages of survivors of pandemic (H1N1) 2009 infection did not differ significantly by sex.

## Conclusions

In this study from the United Arab Emirates, we report the epidemiologic and clinical features of pandemic (H1N1) 2009 infection in Abu Dhabi. The characteristics are similar to those reported in other parts of the world (*10**–**12*). Children were most at risk for pandemic (H1N1) 2009 infection; older adults (>60 years) appeared to be least affected, probably because of cross-protective immunity from exposure to antigenically related influenza viruses earlier in life (*13**,**14*). Twenty-six persons died, most of whom were 21–60 years of age (*7**,**10*). This number translates to an overall incidence of pandemic (H1N1) 2009–associated death in Abu Dhabi of 1.4/100,000 population, which is relatively low compared with some studies (*11**,**15*).

Our findings are subject to limitations. For example, like most epidemiologic studies based on surveillance systems, data are often incomplete, and therefore resulting analysis can be subject to bias. Nonetheless, we believe the aggressive approach implemented by the Abu Dhabi government (e.g., body temperature scans at airports, isolation of persons suspected to have pandemic [H1N1] 2009, tracing of contacts of persons with confirmed cases, and providing oseltamivir prophylaxis) played an important role, not only in delaying the onset and spread of pandemic (H1N1) 2009, but also in reducing deaths.
